# Gene flow on ice: the role of sea ice and whaling in shaping Holarctic genetic diversity and population differentiation in bowhead whales (*Balaena mysticetus*)

**DOI:** 10.1002/ece3.397

**Published:** 2012-10-18

**Authors:** S Elizabeth Alter, Howard C Rosenbaum, Lianne D Postma, Peter Whitridge, Cork Gaines, Diana Weber, Mary G Egan, Melissa Lindsay, George Amato, Larry Dueck, Robert L Brownell, Mads-Peter Heide-Jørgensen, Kristin L Laidre, Gisella Caccone, Brittany L Hancock

**Affiliations:** 1 Department of Biology, York College, City University of New York94-20 Guy R. Brewer Blvd, Jamaica, New York, 11415; 2 CUNY Graduate Center, 365 Fifth AvenueNew York city, New York, 10016; 3 American Museum of Natural History, Sackler Institute for Comparative Genomics79th St and Central Park West, New York city, New York, 10024; 4 Wildlife Conservation Society, Global Conservation Program-Ocean Giants Program185th St and Southern Blvd, Bronx, New York, 10460; 5 Fisheries and Oceans Canada, Center and Arctic Region501 University Crescent, Winnipeg, Manitoba, R3T 2N6, Canada; 6 Department of Anthropology and Archaeology, Memorial University of Newfoundland, St. John's, Newfoundland and LabradorA1C 5S7, Canada; 7 Southwest Fisheries Science Center, NMFS/NOAA1352 Lighthouse Ave., Pacific Grove, California, 93950; 8 Greenland Institute of Natural ResourcesP.O. Box 570, Nuuk, 3900, Greenland; 9 Applied Physics Laboratory, Polar Science Center, University of WashingtonWashington; 10 Department of Ecology and Evolutionary Biology, Yale UniversityNew Haven, Connecticut, USA; 11 Southwest Fisheries Science Center, NMFS/NOAA3333 N. Torrey Pines Ct, La Jolla, California, 92037

**Keywords:** Ancient DNA, arctic, cetacean, marine mammal, mitochondrial DNA, whaling

## Abstract

Sea ice is believed to be a major factor shaping gene flow for polar marine organisms, but it remains unclear to what extent it represents a true barrier to dispersal for arctic cetaceans. Bowhead whales are highly adapted to polar sea ice and were targeted by commercial whalers throughout Arctic and subarctic seas for at least four centuries, resulting in severe reductions in most areas. Both changing ice conditions and reductions due to whaling may have affected geographic distribution and genetic diversity throughout their range, but little is known about range-wide genetic structure or whether it differed in the past. This study represents the first examination of genetic diversity and differentiation across all five putative stocks, including Baffin Bay-Davis Strait, Hudson Bay-Foxe Basin, Bering-Beaufort-Chukchi, Okhotsk, and Spitsbergen. We also utilized ancient specimens from Prince Regent Inlet (PRI) in the Canadian Arctic and compared them with modern stocks. Results from analysis of molecular variance and demographic simulations are consistent with recent and high gene flow between Atlantic and Pacific stocks in the recent past. Significant genetic differences between ancient and modern populations suggest PRI harbored unique maternal lineages in the past that have been recently lost, possibly due to loss of habitat during the Little Ice Age and/or whaling. Unexpectedly, samples from this location show a closer genetic relationship with modern Pacific stocks than Atlantic, supporting high gene flow between the central Canadian Arctic and Beaufort Sea over the past millennium despite extremely heavy ice cover over much of this period.

## Introduction

Sea ice is a dominant feature of the polar environment and is thought to shape patterns of genetic isolation in both marine and terrestrial island species (e.g., Geffen et al. 2007). However, for commercially hunted species such as arctic marine mammals, population genetic structure and diversity also reflect the legacy of whaling and sealing (Roman and Palumbi 2003; Alter et al. 2007; Jackson et al. 2008). Large-scale removals over the last three centuries may have altered pre-whaling genetic differences between populations by disrupting patterns of migration to breeding areas (e.g., Alter et al. 2009) or by eliminating distinct populations from areas and allowing colonization by another stock. Despite these uncertainties, genetic differentiation and stock identity remain important issues for managers and policymakers. Understanding the factors that govern stock structure and gene flow, including the interplay between changing sea-ice conditions and the legacy of whaling, is particularly important for Arctic species that are likely to be affected by climate change, increasing oil and gas development, and shipping, such as the bowhead whale (*Balaena mysticetus*). Ongoing dramatic declines in sea-ice extent will likely affect genetic exchange rates in bowhead whales as well as other arctic marine mammals such as beluga and walruses (Laidre et al. 2008; O'Corry-Crowe 2008), but evaluating these changes requires the characterization of genetic patterns prior to significant ice loss.

Bowhead whales are the only large baleen whale to occur in the Arctic year-round and are highly adapted to the arctic environment, with the thickest blubber layer of any mammal and the ability to break ice 30–60-cm thick (Marquette 1986). All bowhead whale populations spend summers in the Arctic, but overwinter in subarctic seas, inhabiting polynyas and the marginal ice zone, following seasonally advancing and retreating ice edges (Moore and Reeves 1993). Climatic variations during the Holocene were dramatic across some parts of the species' range, and changes in sea-ice cover over the past several millennia may have shaped gene flow between stocks. In addition, genetic patterns may have been affected by whaling. This species was targeted heavily by commercial whalers throughout Arctic and subarctic seas beginning in Labrador around 1540 and continuing into the early 20th century (Ross 1993), resulting in moderate to severe reductions in population abundance across its range (Woodby and Botkin 1993). Although these reductions likely affected both the amount and geographic distribution of genetic diversity in bowhead whales, relatively little is known about range-wide genetic structure today or how it may have differed before large-scale commercial whaling.

**Figure 1:**
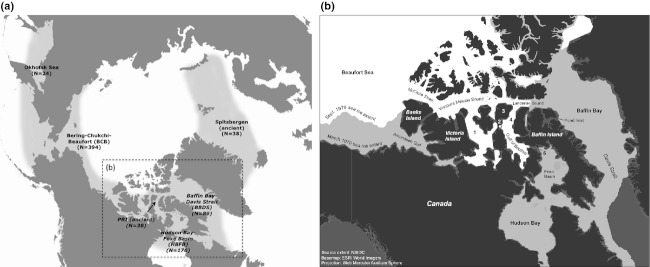
(a) The full range of bowhead whales across the Arctic (lightly shaded area; Laidre et al. 2008) with sample sizes from region. Italicized names represent data collected in this study. Area inside dotted line shows the location of (b) detailed map of the Canadian Arctic. 1 = McClintock Sound, 2 = Qariaraqyak (PaJS-2) (archeological site from which “PRI” samples were excavated), 3 = Somerset Island, 4 = Prince Regent Inlet, 5 = Fury and Hecla Strait. Also shown are maximum (March – lightest gray) and minimum (September – white) sea-ice extent for 1979 (the earliest year data are available from NSIDC).

Bowhead whales have been divided into management stocks largely based on geographic discontinuities, including sea ice perceived as a barrier to movement (Moore and Reeves 1993). Until recently, five stocks of bowhead whale have been recognized by the IWC for management purposes: (1) Hudson Bay-Foxe Basin (“HBFB”); (2) _Baffin Bay-Davis Strait (“BBDS”); (3) Beaufort, Chukchi, and Bering Seas (“BCB”); (4) the Okhotsk Sea (“Okhotsk”); and (5) the area of Spitsbergen and the Barents Sea (“Spitsbergen”) (Fig. 1a). Two separate stocks in Canada and Greenland (HBFB and BBDS) were hypothesized based on the assumption that Fury and Hecla Strait represents a geographic barrier to bowhead whales. Persistent ice plugs throughout the Northwest Passages, which are believed to have been stable from roughly 3 kya until the last several years (Vare et al. 2009), are also thought to prevent migration between BCB and Atlantic stocks. However, recent satellite tracking data show that whales occupying Foxe Basin move through Fury and Hecla Strait into Prince Regent Inlet (PRI), an area that has been traditionally classified as belonging to BBDS (Heide-Jørgensen et al. 2006). This evidence, in combination with abundance data on calves and adults in various areas, suggests that bowhead whales in eastern Canada and Greenland may represent one population (“Canada-Greenland”), rather than two (COSEWIC 2009). Likewise, in 2010, satellite telemetry data demonstrated overlap in movement between a BCB individual and a Canada-Greenland individual in Viscount Melville Sound, which was attributed to the recent and dramatic loss of sea ice in the Canadian Arctic (Heide-Jørgensen et al. 2011).

Despite these observations, questions remain about range-wide population structure among bowhead whales. In particular, the degree and timing of genetic exchange between Atlantic (HBFB/BBDS, Spitsbergen) and Pacific (BCB, Okhotsk Sea) populations has yet to be fully elucidated. Interchange between the Atlantic and Pacific Oceans through the central Canadian Arctic was likely possible during the warmer conditions of the early and mid-Holocene, but climatic reconstructions indicate a rapid increase in ice cover around 3 kya that excluded bowhead whales from the central channels of the archipelago (Dyke et al. 1996; Vare et al. 2009). A short period of lower ice cover may have occurred just before the start of the Little Ice Age in the early 15th century, followed by an increase in ice cover in the last 400 years (Vare et al. 2009). Although this climatic history indicates the last significant connection through the Canadian Arctic occurred >3 kya, the ability of bowhead whales to navigate cracks and leads in extremely dense pack ice (>90% cover) suggests the possibility of more recent exchange. A recent genetic study compared microsatellite data from western Arctic whales with whales from a location in the eastern Canadian Arctic and found low but significant differentiation, suggesting a small degree of genetic mixing (Givens et al. 2010). Another study compared mitochondrial haplotypes from late Pleistocene to late Holocene Spitsbergen samples with those from the modern BCB population and found a similar result (low but significant differentiation), although the difference disappeared when only the most recent Spitsbergen samples were used (Borge et al. 2007). While both these analyses suggest that there has been some mixing between the Atlantic and Pacific, no previous study has attempted to estimate the magnitude or timing of the most recent exchange, or has incorporated whaling history into genetic data analysis. Population bottlenecks due to whaling can reduce haplotype diversity and can affect haplotype frequency distributions, leading to apparent spatial structure between bottlenecked populations (Alter et al. 2012). Thus, accounting for whaling history is critical for accurate analysis of population structure in heavily exploited species.

Historical and ancient samples represent a valuable but underutilized source of information about marine mammal responses to both climate change and whaling over long periods. Genetic data from such samples have been used to test hypotheses about population response to climate shifts and hunting in many terrestrial species (e.g., Shapiro et al. 2004; Chan et al. 2005; Dalen et al. 2007), but have been less frequently utilized in marine systems (but see, e.g., de Bruyn et al. 2009). For many cetacean species depleted by whaling and now recovering, data from historical and ancient DNA (herein both referred to as “ancient DNA”) can provide an important point of comparison for determining how stock identity and genetic diversity differed before large-scale commercial whaling began.

The extensive archeological and stranded remains of bowhead whales across the Arctic provide an opportunity to better understand the factors shaping genetic connectivity in bowhead populations. In this study, we used ancient and modern bowhead control region sequences to compare genetic diversity and population differentiation between: (1) all putative modern management stocks, including sequences from Spitsbergen samples aged 30–3,000 years old (Borge et al. 2007); and (2) modern stocks and ancient samples from PRI, located in the central Canadian Arctic. For the latter comparison, we collected data from the mitochondrial D-loop from bowhead specimens from 500 to 800 years old Thule Inuit house ruins at the east coast of Somerset Island (western side of PRI), and compared them with sequences from the five putative stocks (HBFB, BBDS, BCB, Okhotsk, and Spitsbergen). PRI is situated in the modern-day range of BBDS (Fig. 1b), and ancient samples from this locale are ideal for exploring gene flow between the Pacific and Atlantic populations over the last millennium. We used modern and ancient samples to test the following hypotheses, based on the expectation that persistent ice cover is a barrier to genetic exchange: (1) significant differentiation between Atlantic (HBFB, BBDS, Spitsbergen) and Pacific (BCB, Okhotsk) populations; (2) ancient PRI whales are most closely related to the modern BBDS population; and (3) the last genetic exchange between the Pacific and Atlantic sides of the Canadian Arctic occurred during the mid-Holocene (roughly 3 kya). This approach builds upon previous studies of bowhead whale genetic in two respects: first, we utilized samples from across the entire circumpolar range of bowhead whales and include samples from a late Holocene time period; and second, we used demographic modeling in addition to traditional population structure analyses to test the hypotheses above and to incorporate the impacts of whaling on genetic structure.

## Materials and Methods

### Ancient sample collection

Samples of preserved baleen and bone were collected from archeological sites on Somerset Island (western side of PRI) as described in Whitridge (2002). Qariaraqyuk (with the Canadian archeological site designation PaJs-2) is a Classic Thule winter village located on the southeastern tip of Somerset Island, (Savelle and McCartney 1994; Whitridge 1999), and was occupied from about AD 1200–1500. It was likely a major winter residential locus for groups who whaled from nearby PaJs-4 in late summer/early fall (Savelle and Wenzel 2003). The site consists of a row of at least 57 sod winter houses, making it the largest precontact winter village in the Canadian Arctic (Whitridge 2002). Six of the houses were excavated in 1993–1994. The samples included in the present analysis consists of specimens of artifactual baleen, including artifacts (vessels, cordage, toys, etc.), refuse from artifact manufacture, and knotted strands that likely represent the structural lashing from whale bone house frameworks. Calibrated radiocarbon dates on heather (*Cassiope tetragona*), caribou bone (*Rangifer tarandus*), and willow (*Salix* sp.) from the house assemblages of which these samples are a part bracket the occupation of the features between 500 and 800 ybp.

The location of this site on the western side of PRI/Gulf of Boothia is on the summering ground of what would today be considered part of the BBDS stock. However, satellite tracking data also indicate that the area is also used by animals from Foxe Basin (Greenland Institute of Natural Resources, unpublished data). Bowhead whales only visit PRI, which is characterized by heavy ice cover, for about 2 months per year. Solid fast ice coverage in the Canadian Arctic Archipelago during fall, winter, and spring forces all cetaceans to move out into open water or to areas with mobile pack ice (Moore and Reeves 1993). This forces animals into relatively small pockets of inhabitable areas in eastern Hudson Strait, West Greenland and recurrent polynias on the east coast of Baffin Island and in Lancaster Sound.

### Ancient DNA methods and authentication

All extractions were performed in dedicated ancient DNA facilities at the American Museum of Natural History. No modern whale DNA had been extracted and no amplifications had taken place within this facility. All samples were stored in separate airtight plastic bags until use to prevent cross-contamination. Samples were pretreated to remove potential surface contaminants as described in Rosenbaum et al. (1997). Briefly, all materials used were UV-treated prior to use and bone surfaces were cleaned with kimwipes soaked in ethanol, 10% Clorox, and finally RNAase free H20. Bone surfaces were removed using a clean drill bit treated with HCl and UV light.

Subsamples of bone were obtained using a sterilized drill bit to drill a small hole (<0.5-cm diameter, 3–4-mm deep) to generate ∼0.1g of bone powder. Bone powder was then treated to remove any remaining contaminants by soaking in 10% Clorox for 20 min followed by a rinse in sterile H_2_O. Baleen was subsampled following the protocol of Rosenbaum et al. (1997). Samples were incubated at 37°C for several hours to overnight with 1.5-mL 0.5M EDTA pH 8.5 in order to decalcify bone and remove inhibitors from humic acid. Following incubation with EDTA, samples were centrifuged at 10,000 rpm for 5 min and supernatant was removed. We performed an additional rinse with 1-mL H_2_0 in order to reduce the EDTA concentration. To extract DNA from the bone pellet, samples were incubated with 0.5-mL Lifton's buffer and 35 uL of 20 mg/mL proteinase K at 56°C for 50 h. Extraction was completed using standard phenol/chloroform purification and ethanol precipitation procedures (Sambrook et al. 1989).

Amplification conditions are given in Rosenbaum et al. (1997). All amplifications were set up in the ancient DNA facility, but thermal cycling was carried out in a separate post-extraction lab. A series of primer pairs that generate overlapping fragments of the mitochondrial D-loop were used, including primers Dlp 1.5 and Dlp 5 that amplify the majority of the variable sites in the cetacean D-loop (Arnason et al. 1993; Baker et al. 1993) and six additional primers detailed in Rosenbaum et al. (1997) that amplify 100–200-bp regions. Three bowhead-specific primers were developed for sequencing (Myst3.3A, Bm96f, and Bm218f; available from authors upon request).

Successful amplification products were sequenced in both directions using fluorescence-labeled dideoxy terminators on an ABI 3700 High-throughput Capillary DNA Sequencer (Applied Biosystems). To authenticate sequences, a subset (∼15%) of samples with unique haplotypes were re-extracted, amplified, and sequenced in both directions in an entirely separate facility (a dedicated ancient DNA facility at Yale University, Department of Ecology and Evolutionary Biology), and scored blind relative to the original sequences.

### Modern sequences

Modern D-loop data were generated from biopsy samples collected from areas of Northern Canada and West Greenland including Pelly Bay, Repulse Bay, and Igloolik (previously included in the HBFB stock, *N* = 176) and Disko Bay, West Greenland and Pangnirtung, Canada (previously included in the BBDS stock, *N* = 89) collected between 1997 and 2006. Total cellular DNA was extracted from bowhead skin samples using different techniques. Earlier samples (before 2000) were extracted using the methods described in Maiers et al. (1996) with some modifications. The skin tissue was incubated at 37°C for an extended period and had several additions of proteinase K (20 mg/mL) to digest the tissue to the point where it was suitable for extraction. Once this process was complete, in most samples, sufficient quantities of DNA were recovered for analyses. More recent samples (after 2000) were extracted using commercial DNA tissue extraction kits (DNeasy, Qiagen).

A portion of the mitochondrial DNA D-loop was amplified using primers Dlp 1.5 and Dlp 5 that amplify the majority of variable sites in cetaceans (Arnason et al. 1993; Rosenbaum et al. 2002). Automated DNA sequencing of the PCR products was performed using ABI genetic analyzers (Prism 377, 3100, 3130XL) and the related fluorescent dye terminator chemistry. Samples were also genetically profiled at 21 microsatellite loci (using methods described in Givens et al. 2010). These data were used to detect the occurrence of individuals represented multiple times in the dataset due to recapture of animals. Probability of Identity was assessed using the program GeneCap (Wilberg and Dreher 2004). GeneCap is designed to identify matches, but will also flag samples that match at all alleles, but one or two (which may represent true replicates that were undetected due to genotyping errors).

In addition to generating ancient and modern sequences from the Canadian Arctic, we also utilized previously collected D-loop data from the following populations of bowhead whales: (1) BCB Seas (*N* = 394) (LeDuc et al. 2009); (2) Okhotsk Sea (*N* = 24) (LeDuc et al. 2009); and (3) Spitsbergen sequences from 30 to 3,000 years in age (*N* = 38) (Borge et al. 2007).

### Genetic analysis

Sequences were cleaned and edited using Sequencher v. 4.0 (GeneCodes), and species identity was determined using the NCBI database (BLAST), as well as a diagnostic character approach for species delimitation (Rosenbaum et al. 2000). Haplotypes from ancient samples were compared with sequences from independent extractions/amplifications to assure sequence authenticity. We examined haplotype frequency distributions within and among ancient and modern populations. Genetic diversity was compared among populations using several measures including haplotype diversity (Hd), nucleotide diversity (π), and sequence diversity (θ[S]), generated using DnaSP v.4.0 (Rozas et al. 2003). Partitioning of genetic variation among sample sets was assessed using analysis of molecular variance (AMOVA [analysis of molecular variance]; Excoffier et al. 1992) generated in ARLEQUIN v2.0 (Schneider et al. 2000). Because of the high substitution rate of mtDNA and incorporation of ancient samples, we expect that both genetic drift and mutation are potentially influencing genetic differentiation, and therefore the *Φ*_ST_ statistic is a more appropriate measure of differentiation than frequency-based *F*_ST_ (which does not take into account molecular distances between haplotypes) (Excoffier 2003; Holsinger and Weir 2009). We assessed a priori geographic stratifications based on four (combining HBFB and BBDS into “Canada-Greenland”) or five putative stocks, and also assessed all Pacific (BCB, Okhotsk) versus all Atlantic (HBFB, BBDS, Spitsbergen) populations. Statistical significance of *Φ*_ST_ values in pairwise population comparisons was determined using 10,000 random permutations of the data matrix variables and a Jukes-Cantor evolutionary model (Jukes and Cantor 1969). In addition, we generated an optimal minimum spanning network using Arlequin v2.0 in order to assess the geographic distribution of haplotypes. Optimal minimum spanning networks utilize haplotype frequency data to determine the most parsimonious relationships between haplotypes.

### Demographic simulations

**Figure A1:**
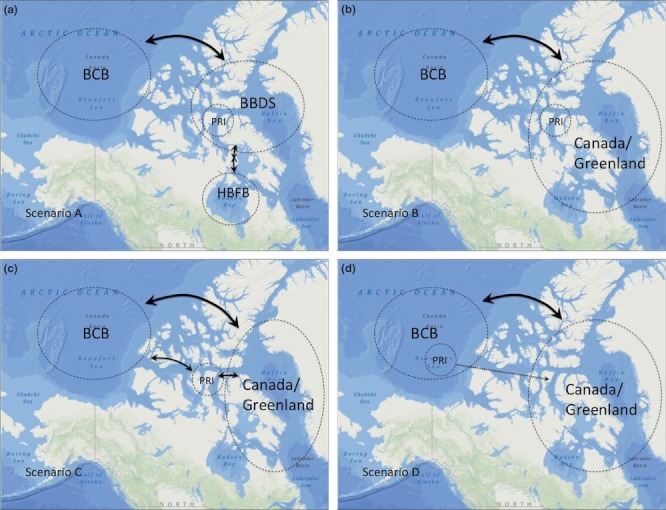
Cartoon representation of demographic scenarios used in simulations (see text). [Boundaries shown below are intended to convey the general geographic location for putative populations, and are not intended in any way to represent the shape or extent of the range of those populations.]

We used a demographic simulation approach to explore whether geographic barriers and whaling may have influenced observed patterns of differentiation. Specifically, we used simulations to determine the expected genetic differentiation between sample sets under scenarios of particular climatic (ice cover) and whaling histories. We used the program BayesSSC (Bayesian Serial SIMCOAL, Anderson et al. 2005) to model demographic scenarios focused on the four sample sets collected in or near the Canadian Arctic (both Atlantic and Pacific): PRI, BBDS, HBFB, and BCB. Other approaches such as IMa (Hey and Nielsen 2007) were considered, but not deemed appropriate for this analysis because of the need to specify samples collected at different time points. The Okhotsk and Spitsbergen populations were not included because these populations are geographically removed from the Canadian Arctic and considerably less is known regarding demographic and whaling histories in these locations. We modeled a total of 36 demographic scenarios between the Atlantic and Pacific. Within each of three basic scenarios (described below), we modeled four subscenarios describing different possibilities for between-stock structure, and three migration rates (*m* = 0.1, *m* = 0.01, *m* = 0.001). In brief, we modeled the following scenarios (see Appendix, Figure A1, Table 3) and residual size as given by Woodby and Botkin (1993), HBFB is reduced to 1–68% of its initial size (Woodby and Botkin 1993), and the BBDS population is reduced to 1–29% of its initial size (Woodby and Botkin 1993). Generation time was assumed to be 52 years in the simulations (Taylor et al. 2007). Simulations were performed using female effective population sizes, which was calculated from census population sizes assuming a 1:1 male:female ratio, 1.5:1 ratio of all individuals to all adults, and an Ne/N ratio of 0.5 (Roman and Palumbi 2003). We used a uniform prior on mutation rate ranging from 2% per my, the fossil-calibrated phylogenetic rate (Roman and Palumbi 2003), to 6.3% per my, which represents the highest rate for baleen whale control region calculated from calibrations using cytochrome*-b* (Alter and Palumbi 2009), and a mutation model (HKY+G) based on results from MODELTEST (Posada and Crandall 1998). Using these parameters, 10,000 independent genetic datasets were simulated per scenario. We determined whether the simulation results were compatible with the observed genetic difference between ancient samples from PRI and modern samples from BBDS and BCB by sampling the simulated datasets to obtain samples of the same size and age as our empirical datasets. Observed *Φ*_ST_ values between BCB versus BBDS, BCB versus Canada-Greenland, BCB versus PRI, PRI versus BBDS, and PRI versus Canada-Greenland were compared to the distribution of *Φ*_ST_ values between the corresponding simulated datasets. If any of the observed pairwise *Φ*_ST_ values fell outside of the 95% highest posterior density interval of the distribution from simulated datasets, the corresponding demographic scenario was rejected.

## Results

### Genetic diversity

We obtained sequence data for 38 ancient samples from PRI and 265 modern samples from HBFB and BBDS (Genbank Accession numbers are provided in the Appendix, Table A2). Once aligned with sequences from BCB, Okhotsk, and Spitsbergen, the complete dataset comprised 370 bp of mitochondrial D-loop for a total of 759 samples (Table 1). Probability of Identity (Wilberg and Dreher 2004) was tested for all HBFB and BBDS samples and was found to be sufficient to permit discrimination of individuals (PID _HW_ = 8.1 × 10^−31^; PID _SIB_ = 1.9 × 10^−10^). Six duplicated sequences were found (4 in HBFB and 2 in BBDS), and removed from the mtDNA sequence set. For ancient samples, no sequence differences were found between original sequences and samples that were re-extracted and sequenced in an independent facility. Haplotype, nucleotide, and sequence diversity were high for all sample sets examined, with the exception of Okhotsk Sea. Haplotype diversity was significantly higher in PRI (95% confidence intervals: 0.860−0.977) and BCB (0.892−0.935) compared with Canada-Greenland (0.785−0.848) and Okhotsk (0.484−0.775), based on coalescent analyses performed in DNAsp (Rozas et al. 2003).

**Table 1:** Number of samples for each sample set (*N*) and diversity values across populations

	*N*	*S*	*H*	*Hd*	*U*	*π*	*θ(S)*
PRI (Ancient)	38	26	20	0.92	7	0.014	7.62
Canada-Greenland	265	26	23	0.8	6	0.007	5.2
HBFB	176	25	20	0.81	4	0.007	5.4
BBDS	89	22	15	0.79	2	0.008	5.34
BCB	394	36	54	0.9	15	0.01	6.41
Okhotsk	24	24	4	0.63	0	0.007	3.21
Spitsbergen (Ancient)	38	19	17	0.85	8	0.008	5.24

S, number of segregating sites; H, number of haplotypes; Hd, haplotype diversity; U, unique haplotypes; π, nucleotide diversity; θ(S) based on segregating sites. Canada-Greenland, HBFB and BBDS combined.

Across all samples, a total of 76 haplotypes were observed for an overall haplotype diversity of 0.87. Private haplotypes were observed in all populations with the exception of Okhotsk, and the majority (67%) of these private haplotypes were singletons in the dataset. The most frequently observed haplotype was the same for all sample sets, and the second most frequently observed haplotype was the same for five of the six sample sets (with Okhotsk being the exception).

### Population differentiation

Among-population variation accounted for 1.68% of total variation, compared with 0.27% for the between-group (Atlantic and Pacific) comparison (Table 2a). No differentiation was observed between HBFB and BBDS (*Φ*_ST_ = −0.002, *P* > 0.1) (See Appendix, Table A3), and these two populations were subsequently grouped together as Canada-Greenland. However, we observed significant Φ_ST_ values for most other pairwise population comparisons (Table 2b). The analysis revealed a small but significant amount of differentiation between BCB and Canada-Greenland, and between BCB and PRI. Larger *Φ*_ST_ values were estimated between Okhotsk and all other sample sets, and between PRI and Canada-Greenland. All significant *Φ*_ST_ values remained significant after a False Discovery Rate correction for multiple comparisons (Benjamini and Hochberg 1995) was applied.

**Table 2:** (a) Hierarchical AMOVA results, including variation between two groups of putative populations (Pacific and Atlantic), among putative populations, and within populations. (b) Pairwise genetic distances between sample sets: pairwise *Φ*_ST_ values are given below the diagonal and frequency-based *F*_ST_ values are given above. All bold values are significant after a False Discovery Rate correction

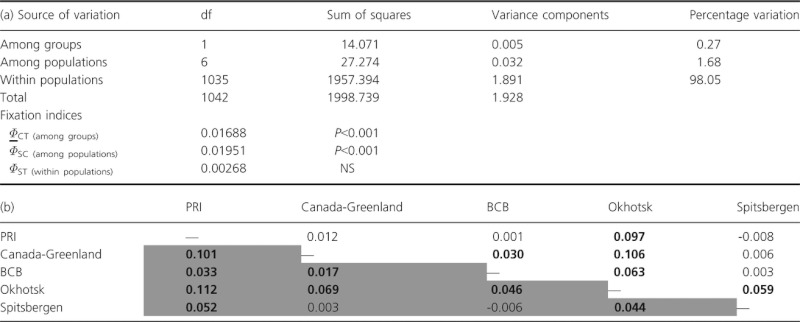

**Figure 2:**
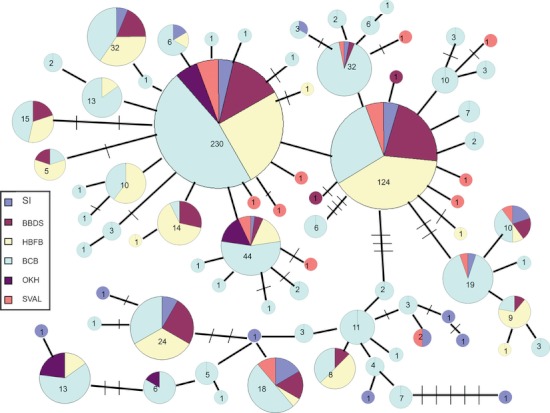
Minimum spanning network for bowhead whale haplotypes created using TCS. Size of circle is proportional to number of samples; hatchmarks represent additional segregating sites.

The optimal minimum spanning network (Fig. 2) shows the geographic distribution of haplotypes as well as their frequencies, demonstrating a weak geographic signal overall. The most frequently observed haplotypes are distributed widely throughout the Arctic. A large number of singletons are observed for BCB and the two ancient sample sets (PRI and Spitsbergen).

### Demographic simulations

**Figure 3:**
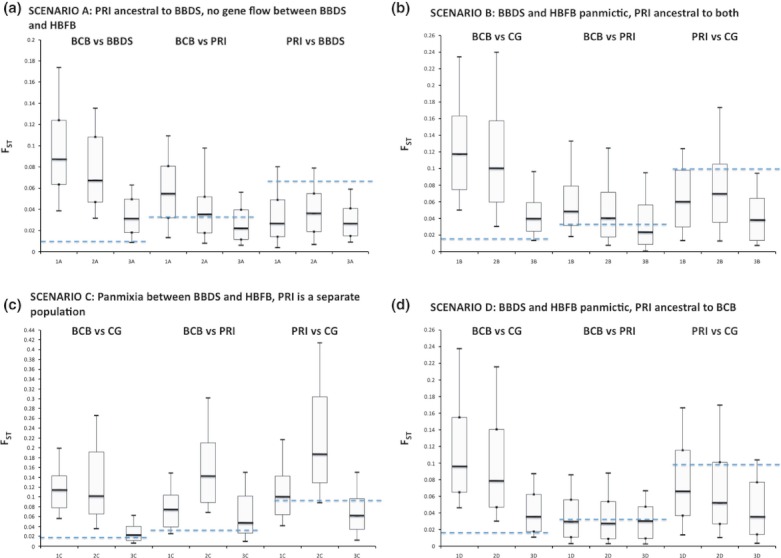
Simulated and empirical pairwise *Φ*ST values between putative populations for *m* = 0.1. Vertical lines show 95% highest posterior density (HPD) intervals for simulated *Φ*ST values; top and bottom of box indicate 75% HPD intervals and thick horizontal lines show median values for simulations. Dotted horizontal lines show empirically observed pairwise *Φ*ST values. CG = Canada-Greenland (BBDS and HBFB combined). (a) Scenario A; (b) Scenario B; (c) Scenario C; (d) Scenario D (see text for details).

As no genetic differentiation was observed between BBDS and HBFB (see above), we grouped these two stocks into a single population (Canada-Greenland) for the majority of scenarios. The distribution of *Φ*_ST_ values for three pairwise comparisons across 10,000 simulations (*m* = 0.1) is shown in Figure 3. We compared these simulated distributions with empirically observed *Φ*_ST_ values (Table 2). For the comparison between BCB and either BBDS alone (Scenario A) or Canada-Greenland, the observed value of *Φ*_ST_ falls within the 95% highest posterior density (HPD) interval for 3A, 3B, 3C, and 3D at *m* = 0.1. For the BCB-PRI comparison, the observed value of *Φ*_ST_ falls within the 95% HPD interval for all scenarios that use *m* = 0.1 with the exception of 2C. For the comparison between PRI and BBDS alone or Canada-Greenland, the observed *Φ*_ST_ falls within the 95% HPD interval for 1A, 2A, 2B, 1C, 3C, and 3D at *m* = 0.1. Based on these results, the following scenarios can be excluded (e.g., the range of *Φ*_ST_ values does not include the observed value): 1A, 2A, 3A, 1B, 2B, 3B, 1C, 2C, 1D, and 2D. In other words, for *m* = 0.1, the only scenarios that are not excluded are 3C (contemporary gene flow with PRI as a separate population) and 3D (contemporary gene flow with PRI ancestral to BCB). For lower migration rates (*m* = 0.01 and *m* = 0.001), all scenarios are excluded (see Appendix, Table A4).

## Discussion

Our analysis of the five putative populations of bowhead whales, including data from ancient samples from two locations (PRI and Spitsbergen), represents the first genetic comparison across the entire range of the species and illustrates the utility of ancient specimens in reconstructing the history of genetic exchange in exploited marine mammals. The results indicate that Arctic sea ice has not acted as a strong barrier to migration between the Atlantic and Pacific over the late Holocene as previously assumed, and that genetic diversity has been lost from eastern Canada in the period between ∼500 ybp and the present.

### Genetic diversity

Measures of modern diversity are similar to those reported in earlier studies of population-level diversity in the control region of bowhead whales (e.g., LeDuc et al. 2005, 2009; Borge et al. 2007). However, in contrast to Borge et al. (2007)'s finding that genetic diversity was similar between ancient whales from Spitsbergen and modern BCB population, genetic diversity (including Hd, π and θ(S)) in ancient samples from PRI is significantly higher compared with modern Atlantic populations, providing a measure of lost haplotypic diversity over the last millennium (Table 1). Diversity in modern sample sets is broadly consistent with relative sizes of populations: BCB shows the greatest amount of diversity, and Okhotsk the least. These results agree with earlier studies that observed low diversity (7 haplotypes across 67 individuals) in the Sea of Okhotsk (MacLean 2002; LeDuc et al. 2009).

The high number of mitochondrial haplotypes observed across bowhead whale populations may not be atypical for baleen whales. Jackson et al. (2008) observed 38 haplotypes in the southern right whale (*Eubalaena australis*) population, and estimated pre-exploitation richness at 100–150 haplotypes. We observed 62 haplotypes in the modern dataset alone, without any correction for undersampling or sequence length, which would increase the number of estimated haplotypes.

The relatively high number of unique or population-specific haplotypes among PRI samples (roughly one-fifth of PRI haplotypes are unique to that sample set), as well as the high divergence of several of these haplotypes compared with modern samples (Fig. 2), suggests that lineage diversity in the Canadian Arctic was greater as recently as 500 ybp. This observation represents the first empirical demonstration of lost haplotype diversity in bowhead whales over the last several centuries. Potential causes for this loss, including whaling, are explored below.

### Genetic differentiation and timing of exchange between Atlantic and Pacific

In contrast with previous hypotheses about connectivity between BCB and Atlantic populations based on subfossils and sea-ice reconstructions (e.g., Moore and Reeves 1993; Dyke et al. 1996; Vare et al. 2009), AMOVA and simulation results are most consistent with contemporary and high gene flow between the two ocean basins. The degree of differentiation estimated between BCB and Atlantic populations is in agreement with the slight but significant allelic differentiation (*F*_st_ = 0.009) between Barrow, Alaska (*n* = 231) and Igloolik, Canada (*n* = 37) estimated using 21 microsatellite loci (Givens et al. 2010). These results contrast markedly with estimated divergence times between North Atlantic and North Pacific populations in other whale species (fin whales, 1.05–2.70 Mya (Berube et al. 1998); common minke whales, ∼1.5 Mya (Pastene et al. 2007); humpback whales ∼2–3 Mya (Baker et al. 1993; right whales, >3.5 Mya (Rosenbaum et al. 2000)), and underscore the adaptation of bowhead whales to arctic habitat relative to other baleen whales.

Within ocean basins, we did not observe any consistent relationship between ice barriers and population differentiation. Although BBDS and HBFB have previously been considered separate management stocks based on the assumption that persistent ice in Fury and Hecla Strait would prevent movement between them, we found no significant differentiation between them. These results are consistent with observed movement patterns from satellite telemetry data, but need to be tested with additional markers, which was not possible in the scope of this study.

### Ancient samples: relationship of PRI and Spitsbergen to modern populations

Severe population reductions due to whaling may have altered signals of genetic diversity and connectivity between bowhead populations, making it difficult to reconstruct pre-whaling patterns. Data from ancient PRI samples can provide insights into how population structure differed in the past. PRI is located in the summering ground of the modern Canada-Greenland population, but has been connected to the Beaufort Sea (BCB population) via the Northwest Passages intermittently over the Holocene. The data presented here show that the genetic difference between PRI samples from 500 to 800 ybp and modern Canada-Greenland samples is unexpectedly larger than the difference between these ancient samples and modern BCB samples.

If PRI whales were the forbearers of modern Canada-Greenland, as geography would suggest, what could cause such strong genetic differentiation between them? One possibility is that the population bottleneck during the height of whaling caused a dramatic reshuffling of haplotype frequencies between the two sampling time points. However, simulations show that even an extreme (∼71–99%) reduction in the Canada-Greenland population does not explain the degree of genetic differentiation between the two. An alternative explanation, which is supported by simulation results (Scenario C) is that PRI samples represent a genetically unique population or set of maternal lineages based around site fidelity to a summering ground on the western side of the Inlet. Recent observations suggest that PRI is a summering area for female whales with calves and juveniles that move from Foxe Basin in the early summer (Dueck and Ferguson 2008), as well as many subadult and adult whales that spend the spring in Baffin Bay and Davis Strait. Intergenerational fidelity to important habitat has been demonstrated in gray whales (Alter et al. 2009), humpback whales (Baker et al. 1994; Palsbøll et al. 1995), right whales (Schaeff et al. 1993), beluga whales (Brown Gladden et al. 1997), and sperm whales (Lyrholm et al. 1999), and other authors have speculated that fidelity to calving grounds in bowhead whales may also be “behaviorally rigid” (Dueck and Ferguson 2008). McCartney (1979) estimated that whaling sites on the western side of PRI contain on the order of 40% of all archeological whale bone across the Canadian Arctic, suggesting that this region was also important as a summering ground in the past, and whalers referred to PRI as a “nursery ground” (Finley 1990).

While median simulated *Φ*_ST_ values are consistent with PRI as an independent population (Scenario 3C), simulation results support high exchange between PRI and BCB, and did not exclude full panmixia between them (Scenario 3D). These findings support the idea that exchange across the Canadian Arctic was occurring during the late Holocene, prior to the recent decrease in sea ice that has permitted overlap in range between Atlantic and Pacific populations (Heide-Jørgensen et al. 2011).

An important consideration in evaluating these results is that we were unable to confirm that each ancient sample represents a unique individual because of difficulties genotyping ancient samples using microsatellites. The high number of unique haplotypes among PRI individuals (Hd = 0.92) and the large number of bowhead individuals recovered from the PaJs-2 locality (a minimum of 261 animals site-wide, not including buried bone, which comprised a significant proportion of remains at the site, minimum number of individuals based on same-side proximal mandible counts (Whitridge 2002)) suggest that most, if not all, of the samples represent different individuals. However, any individuals represented twice or more could spuriously reduce the genetic diversity estimate for PRI, and could result in different estimates of genetic differentiation between PRI and other populations. We tested the impact of 1–5 repeated individuals by serially removing repeated sequences from the dataset and recalculating *Φ*_ST_ values (Appendix, Table A5). Values changed by only −0.014% on average for one duplicated individual and by + 0.036% for five duplicated individuals, suggesting that the impact of duplication on the analysis at this level should be low.

The strong differentiation between PRI and modern populations based on AMOVA results contrasts with overall lack of differentiation between the other set of ancient samples, Spitsbergen, with modern populations (with the exception of the comparison with Okhotsk). No differentiation was observed between BCB and Spitsbergen, corroborating earlier findings by Borge et al. (2007) based on fewer BCB samples compared with only the 25 youngest Spitsbergen samples. This, in combination with significant differentiation between BCB versus PRI and BCB versus Canada-Greenland, presents the possibility that gene flow between the Atlantic and Pacific may have occurred clockwise or westward through the East Siberian and Laptev Seas, rather than (or in addition to) through the Canadian Arctic. These results also suggest a contrast between Spitsbergen as a relatively large and demographically open population spread across a wide geographic area (perhaps similar to BCB today), versus PRI, which is located in a geographic cul-de-sac and which does not reflect range-wide genetic diversity to the same degree as Spitsbergen. However, all comparisons using Spitsbergen samples must be interpreted with caution, as noted by Borge et al. (2007), as the temporal spread of sample ages could potentially introduce spurious genetic diversity in the context of a spatial analysis.

### The role of climate changes, whaling, and ice entrapment in the extirpation of ancient PRI lineages

What might have caused the disappearance of PRI haplotypes from the modern populations of bowhead whales? Both changing climate at the end of the Holocene and whaling (and perhaps an interaction between the two) may have played a role. The most obvious and significant source of mortality for bowhead whales between 500 ybp and the present was commercial whaling, which eliminated a large part of the population (Woodby and Botkin 1993). By far, the largest proportion of the bowhead catches were taken in Davis Strait and Baffin Bay, and whalers were only capable of entering the dense sea ice of northern PRI late in the whaling period (Ross 1993). No whaling records exist to suggest that commercial whalers visited southern PRI. However, commercial whaling in the Central Canadian Arctic coincided with the end of the Little Ice Age (100–400 ybp), a period of much cooler temperatures that marked the sudden disappearance of Thule Inuit settlements from PRI (Savelle and McCartney 1994). This period of climatic cooling likely resulted in an increase in summer sea ice in PRI and the partial or complete loss of this habitat as an important summering ground for bowheads. Animals that used southern PRI would have been forced to use other, potentially suboptimal or already inhabited, summering areas. These new summering areas, which were likely farther east, may have brought more whales into contact with commercial whaling. Moore and Reeves (1993) note the possibility that during the Little Ice Age, “the bowhead population probably experienced restricted access to the summer feeding range while at the same time being intensively exploited by commercial whalers.” The “west water” fishery targeting the areas around Pond Inlet, Lancaster Sound, PRI and northern Gulf of Boothia began in 1827 (Ross 1993), and the next few decades represent the highest removal rates by far across the entire history of bowhead whaling in eastern Canada/western Greenland (Higdon 2010). Whalers documented how in heavy ice years, land-floe ice blocked the entrances to Pond Inlet and Lancaster Sound and whales were unable to migrate further west (summarized in Higdon 2008). Whalers took large numbers of whales during these “closed seasons” when whales would concentrate along the land floe. Thus, the interplay of climate fluctuations and whaling may have played a role in the loss of genetic diversity in the Canadian Arctic.

Two other explanations for the disappearance of PRI haplotypes are possible (and are not mutually exclusive with each other or the hypothesis of climate change/commercial whaling): (1) Thule Inuit whaling; and (2) ice entrapment. Extensive archeological surveys of Thule sites resulted in minimum estimates of 1,830–2,745 whales from Somerset Island alone (McCartney 1979; summarized in Stoker and Krupnik 1993). These numbers represent catches over a period of 300–400 years, so annual takes would likely have been on the order of 10 or fewer whales. Stoker and Krupnik (1993) estimated eight whales taken per year for Somerset Island. However, Higdon (2010) notes that these numbers may be severe underestimates, as buried bones were not included in these original analyses. Sea-ice entrapments represent another potential source of mass mortality for bowhead whales. The inner Canadian Arctic Archipelago including PRI has severe ice conditions in late autumn and whales that depart late can become entrapped and die. There was a report of a bowhead whale entrapped in Lancaster Sound in 1999 and of narwhals entrapped in the southern part of PRI in 1979 (Heide-Jorgensen et al. 2002). Of all areas where bowhead whales concentrate in large numbers, PRI is undoubtedly the most dangerous area where large-scale entrapments could occur. None of the other areas are so far from open water, mobile pack ice, or recurrent polynias necessary for survival in extreme ice. One very large event, or perhaps several large-scale entrapments over a period of time, could potentially explain the historical loss of genetic diversity in PRI. These possibilities demonstrate the broad range of ecological and anthropogenic forces that could have impacted the distribution of genetic diversity in bowhead whales in the Canadian Arctic and beyond.

## Conclusions

These results highlight the complex interplay of factors – including climate history, behavioral ecology, and past exploitation – that shape population genetic patterns in polar marine mammals. In contrast with our initial hypothesis that Pacific and Atlantic populations were last connected during the mid-Holocene (e.g., Dyke et al. 1996), the genetic data presented here instead indicate recent and high gene flow between these areas. At the Holarctic scale, these results suggest that the presence of persistent sea ice does not appear to be a good predictor of genetic exchange in bowhead whales. This finding underscores earlier observations that apparent geographic barriers are not always accurate indicators of population structure in cetaceans (Hoelzel 1994, 1998).

We have attempted to infer a complex demographic history from limited genetic data, and additional genetic information and ancient samples from throughout the region will be needed to further test these hypotheses. Nevertheless, the unique set of maternal lineages found in the central Canadian Arctic (PRI) and the unexpected relationships between this area and other modern populations demonstrate the value of ancient samples in better understanding the role of climatic history and human hunting in shaping genetic diversity and structure in arctic species. Additional ancient samples from across the range of the bowhead whale and integrated data from SNPs and microsatellites would advance analyses beyond the rough estimates possible using mitochondrial data alone. Such studies would allow an unprecedented evaluation of both natural and anthropogenic impacts on genetic variability in a key indicator species in the rapidly changing Arctic.

## Appendix

**Table A1:** Parameters used in demographic simulations

Parameter	Range of values	Distribution	Citations
Mutation rate	2–6.3% per my	Uniform	Roman and Palumbi 2003; Alter and Palumbi 2009;
Generation time	52 years	NA	Taylor et al. 2007;
Length of sequence	370 bp	NA	NA
Initial size (BCB)	13854 (9466–28475)	Normal	Brandon and Wade 2006;
Initial size (BBDS)	10369 (10333–11,000)	Normal	Woodby and Botkin 1993; Table 10.3 and 10.4
Initial size (HBFB)	452 (445–680)	Normal	Woodby and Botkin 1993; Table 10.3 and 10.5
Residual population (BCB)	1000 (100–3000)	Normal	Woodby and Botkin 1993; Table 10.3
Residual population (BBDS)	1000 (100–3000)	Normal	Woodby and Botkin 1993; Table 10.3
Residual population (HBFB)	100 (10–300)	Normal	Woodby and Botkin 1993; Table 10.3
Current size (BCB)	10,470 (8100–13,500)	Normal	George et al. 2004;
Current size (BBDS)	6344 (3119–12916)	Normal	COSEWIC 2009;
Current size (HBFB)	1525 (333–6990)	Normal	COSEWIC 2009

**Table A2:** Haplotype names and Genbank Accession numbers. PRI = number of individuals with Hap_1, etc. found in PRI. ECA = number of individuals with Hap_1, etc. found in ECA

Haplotype number	PRI	ECA	Genbank Accession #
1	6	76	JX507921
2	9	87	JX507922
3	3	4	JX507923
4	2	14	JX507924
5	1	9	JX507925
6	2	17	JX507926
7	0	13	JX507927
8	2	3	JX507928
9	1	1	JX507929
10	0	5	JX507930
11	0	8	JX507931
12	0	4	JX507932
13	0	1	JX507933
14	0	1	JX507934
15	0	7	JX507935
16	0	6	JX507936
17	0	2	JX507937
18	1	1	JX507938
19	0	1	JX507939
20	0	2	JX507940
21	0	1	JX507941
22	0	1	JX507942
23	1	0	JX507943
24	1	0	JX507944
25	1	0	JX507945
26	1	0	JX507946
27	1	0	JX507947
28	1	0	JX507948
29	1	0	JX507949
30	1	0	JX507950
31	1	0	JX507951
32	1	0	JX507952
33	1	0	JX507953
34	1	0	JX507954

**Table A3:** Pairwise genetic distances between sample sets assuming five stocks. Pairwise *Φ*_ST_ values are given below the diagonal and frequency-based *F*_ST_ are given above. All bold values are significant at *P* < 0.05 after a False Discovery correction and bold values in italics are significant at *P* < 0.001

**Table A4:** (a) Distribution of *Φ*_ST_ values for all scenarios using a migration value of *m* = 0.01. (b) Distribution of *Φ*_ST_ values for all scenarios using a migration value of *m* = 0.001

(a)									
	BCB-BBDS			BCB-PRI			PRI-BBDS		
			
SCENARIO	1A	2A	3A	1A	2A	3A	1A	2A	3A
1% HPD	0.057	0.041	0.033	0.032	0.014	0.016	0.000	0.004	0.003
MEDIAN	0.107	0.082	0.078	0.073	0.042	0.053	0.030	0.030	0.040
99% HPD	0.202	0.127	0.153	0.135	0.090	0.110	0.080	0.084	0.078

	BCB-Canada/Greenland			BCB-PRI			PRI-Canada/Greenland		
			
	1B	2B	3B	1B	2B	3B	1B	2B	3B
1% HPD	0.069	0.054	0.048	0.012	0.011	0.011	0.039	0.032	0.031
MEDIAN	0.127	0.102	0.095	0.031	0.029	0.042	0.085	0.067	0.072
99% HPD	0.246	0.209	0.207	0.072	0.060	0.079	0.227	0.141	0.185

	1C	2C	3C	1C	2C	3C	1C	2C	3C
1% HPD	0.058	0.109	0.031	0.022	0.094	0.014	0.039	0.124	0.016
MEDIAN	0.110	0.207	0.081	0.072	0.187	0.050	0.089	0.248	0.062
99% HPD	0.194	0.347	0.185	0.163	0.378	0.117	0.191	0.515	0.201

	1D	2D	3D	1D	2D	3D	1D	2D	3D
1% HPD	0.054	0.042	0.037	0.007	0.006	0.003	0.027	0.023	0.020
MEDIAN	0.127	0.102	0.095	0.031	0.029	0.042	0.085	0.067	0.072
99% HPD	0.276	0.236	0.235	0.082	0.067	0.088	0.262	0.159	0.213

(b)									
	BCB-BBDS			BCB-PRI			PRI-BBDS		
			
SCENARIO	1A	2A	3A	1A	2A	3A	1A	2A	3A
1% HPD	0.061	0.065	0.056	0.040	0.048	0.043	0.002	0.000	0.000
MEDIAN	0.122	0.116	0.135	0.095	0.087	0.103	0.034	0.027	0.031
99% HPD	0.268	0.216	0.245	0.166	0.165	0.186	0.081	0.081	0.091

	BCB-Canada/Greenland			BCB-PRI			PRI-Canada/Greenland		
			
	1B	2B	3B	1B	2B	3B	1B	2B	3B
1% HPD	0.075	0.077	0.069	0.046	0.039	0.060	0.013	0.016	0.009
MEDIAN	0.179	0.173	0.173	0.122	0.109	0.142	0.048	0.053	0.051
99% HPD	0.392	0.344	0.368	0.302	0.310	0.310	0.193	0.152	0.132

	1C	2C	3C	1C	2C	3C	1C	2C	3C
1% HPD	0.071	0.100	0.077	0.048	0.066	0.048	0.077	0.117	0.085
MEDIAN	0.159	0.208	0.172	0.117	0.173	0.128	0.176	0.249	0.218
99% HPD	0.348	0.386	0.331	0.255	0.379	0.274	0.412	0.455	0.385

	1D	2D	3D	1D	2D	3D	1D	2D	3D
1% HPD	0.074	0.058	0.064	0.000	0.002	0.004	0.046	0.036	0.053
MEDIAN	0.166	0.150	0.189	0.036	0.023	0.027	0.125	0.111	0.147
99% HPD	0.324	0.281	0.303	0.081	0.074	0.083	0.276	0.237	0.327

**Table A5:** Sequences were serially removed from sample sets. For each population comparison, *Φ*_ST_ was calculated and averaged across all possible combinations of removed samples, and compared with the observed *Φ*_ST_ value

Number of samples removed	Average *Φ*_ST_	Standard error	% change
PRI versus Canada-Greenland			
1	0.105	0.008	−4.2%
2	0.089	0.014	13.5%
3	0.090	0.016	11.1%
4	0.096	0.021	4.6%
5	0.102	0.021	−0.7%
PRI versus BBDS			
1	0.067	0.009	−1.7%
2	0.058	0.010	15.9%
3	0.059	0.013	10.5%
4	0.064	0.016	2.8%
5	0.069	0.016	−3.9%
PRI versus HBFB			
1	0.102	0.025	4.7%
2	0.095	0.014	12.9%
3	0.096	0.016	10.7%
4	0.102	0.021	4.5%
5	0.107	0.021	−0.1%
PRI versus BCB			
1	0.035	0.006	−7.6%
2	0.028	0.008	24.5%
3	0.028	0.010	13.9%
4	0.034	0.013	−2.1%
5	0.035	0.014	−4.8%
PRI versus Okhotsk			
1	0.114	0.006	−1.5%
2	0.098	0.009	8.1%
3	0.098	0.010	12.2%
4	0.101	0.014	9.6%
5	0.104	0.015	7.5%
PRI versus Spitsbergen			
1	0.051	0.028	1.7%
2	0.033	0.008	15.7%
3	0.035	0.011	33.0%
4	0.041	0.016	22.1%
5	0.040	0.013	23.4%
